# *Heme Oxygenase-1 and 2 Common* Genetic Variants and Risk for Multiple Sclerosis

**DOI:** 10.1038/srep20830

**Published:** 2016-02-12

**Authors:** José A. G. Agúndez, Elena García-Martín, Carmen Martínez, Julián Benito-León, Jorge Millán-Pascual, María Díaz-Sánchez, Patricia Calleja, Diana Pisa, Laura Turpín-Fenoll, Hortensia Alonso-Navarro, Pau Pastor, Sara Ortega-Cubero, Lucía Ayuso-Peralta, Dolores Torrecillas, Esteban García-Albea, José Francisco Plaza-Nieto, Félix Javier Jiménez-Jiménez

**Affiliations:** 1 Department of Pharmacology, University of Extremadura, Cáceres, SPAIN; 2 Department of Pharmacology, University of Extremadura, Badajoz, SPAIN; 3 CIBERNED,Centro de Investigación Biomédica en Red de Enfermedades Neurodegenerativas, Instituto de Salud Carlos III, Madrid, SPAIN; 4 Service of Neurology, Hospital Universitario Doce de Octubre, Madrid, SPAIN; 5 Department of Medicine, University Complutense, Madrid, SPAIN; 6 Section of Neurology, Hospital La Mancha-Centro, Alcázar de San Juan (Ciudad Real), SPAIN; 7 Centro de Biología Molecular Severo Ochoa (CSIC-UAM), Facultad de Ciencias, Universidad Autónoma, Cantoblanco, Madrid, SPAIN; 8 Department of Medicine-Neurology, Hospital “Príncipe de Asturias”. Universidad de Alcalá, Alcalá de Henares (Madrid), SPAIN; 9 Section of Neurology, Hospital Universitario del Sureste. Arganda del Rey (Madrid), SPAIN; 10 Neurogenetics Laboratory, Division of Neurosciences, Center for Applied Medical Research, Universidad de Navarra, Pamplona, SPAIN; 11 Department of Neurology, Clínica Universidad de Navarra, University of Navarra School of Medicine, Pamplona, SPAIN; 12 Department of Neurology, Hospital Universitari Mutua de Terrassa, Terrassa, Barcelona, SPAIN

## Abstract

Several neurochemical, neuropathological, and experimental data suggest a possible role of oxidative stress in the ethiopathogenesis of multiple sclerosis(MS). Heme-oxygenases(HMOX) are an important defensive mechanism against oxidative stress, and HMOX1 is overexpressed in the brain and spinal cord of MS patients and in experimental autoimmune encephalomyelitis(EAE). We analyzed whether common polymorphisms affecting the *HMOX1* and *HMOX2* genes are related with the risk to develop MS. We analyzed the distribution of genotypes and allelic frequencies of the *HMOX1* rs2071746, *HMOX1* rs2071747, *HMOX2* rs2270363, and *HMOX2* rs1051308 SNPs, as well as the presence of Copy number variations(CNVs) of these genes in 292 subjects MS and 533 healthy controls, using TaqMan assays. The frequencies of *HMOX2 rs1051308*AA genotype and *HMOX2 rs1051308A* and *HMOX1 rs2071746A* alleles were higher in MS patients than in controls, although only that of the SNP *HMOX2* rs1051308 in men remained as significant after correction for multiple comparisons. None of the studied polymorphisms was related to the age at disease onset or with the MS phenotype. The present study suggests a weak association between *HMOX2* rs1051308 polymorphism and the risk to develop MS in Spanish Caucasian men and a trend towards association between the *HMOX1 rs2071746A* and MS risk.

Despite Genome-wide association studies (GWAS) in MS confirmed more than 100 loci with genome-wide significance (most of them with modest odds-ratio-OR), only HLA (in particular the HLA-DRB1*15:01 haplotype) showed a strong association with the risk for MS^1^. Many studies of oxidative stress markers in the brain, spinal cord, and cerebrospinal fluid of patients diagnosed with MS, and in the experimental model of autoimmune encephalomyelitis (EAE), suggested a possible role of oxidative stress and lipid peroxidation in the pathogenesis of MS (revised in^1^).

Heme oxygenase is an essential enzyme in heme catabolism by cleaving heme to biliverdin, which is subsequently converted to bilirubin and carbon monoxide (CO). Heme oxygenase occurs as 2 main isozymes, an inducible heme oxygenase-1 (HMOX1), and a constitutive heme oxygenase-2 (HMOX2), which are encoded by the genes designated, respectively, as *HMOX1, HO-1* or *HSP32* (gene identity 3162, chromosome 22q13.1) and *HMOX2* or *HO-2* (gene identity 3163, chromosome 16p13.3). Several recent studies have shown association between certain single nucleotide polymorphisms (SNPs) in the *HMOX1*^2^,^3^,^4^,^5^ and *HMOX2*^4^,^6^ genes and the risk for Parkinson’s disease (PD)^2^,^4^,^6^, essential tremor (ET)^5^, and restless legs syndrome^7^.

HMOX1 expression have been found up-regulated both in the brain^8^,^9^,^10^,^11^ and in the spinal cord^11^ of experimental models of EAE, and in the inflammatory lesions in the brain of patients diagnosed with MS^11^,^12^ and acute disseminated leucoencephalomyelitis^11^. Fagone *et al*.^13^ showed decreased expression of HMOX1 in peripheral blood mononuclear cells of MS patients, the decrease being more evident during exacerbations of the disease. A recent study with DNA microarray analysis in leukocytes from the cerebrospinal fluid of MS patients showed decreased expression of the *HMOX1* gene^14^. It has been reported that EAE induced in HMOX1 knockout mice is more severe than in HMOX1 wild-type mice in terms of Central Nervous System demyelination, paralysis, and mortality, being these effects partially reversed by administration cobalt protoporphyrin IX (inductor of HMOX1) or CO^15^. Induction of HMOX1 by haemin can inhibit EAE as well^16^. In addition, the prolonged prophylactic administration of CO-releasing molecules (CORMs) partially improves clinical and histopathological features of EAE in rodent models, and it has been proposed as potentially useful in the treatment of MS^17^,^18^.

HMOX1 acts as a heat shock protein, and is induced by oxidative stress. The possible role of oxidative stress in the pathogenesis of MS^1^, together with the previously mentioned data regarding HMOX1 in MS and in the EAE model makes that analysis of a possible relationship between *HMOX* polymorphisms and MS risk could seem reasonable, despite *HMOX* polymorphisms have not been mentioned among the possible susceptibility genes in GWAS. Recently, Zborníková *et al*.^19^ showed lack of association between *HMOX1* gene promoter (GT)n polymorphism and progression of MS in a sample of 338 MS patients.

With the aim of investigate a possible association between *HMOX1* and *HMOX2* polymorphisms and the risk of developing MS, we genotyped *HMOX1* and *HMOX2* SNPs in a large group of Caucasian Spanish MS patients and controls.

## Results

The frequencies of the *rs2071746, rs2071747, rs2270363,* and *rs1051308* genotypes and allelic variants, both in MS patient and control groups, were in Hardy-Weinberg’s equilibrium (Table 1). With a single exception, all participants carried two copies of the *HMOX1* and *HMOX2* genes; only one control individual carried a single copy of the *HMOX1* gene; and hence CNVs were not further considered as major putative risk factors. The frequencies of *HMOX2 rs1051308*AA genotype and *HMOX2 rs1051308A* and *HMOX1 rs2071746A* alleles were significantly higher in MS patients than in controls (Table 2). *HMOX2 rs1051308*AA genotype and *HMOX2 rs1051308A* allele were overrepresented in males, and *HMOX1 rs2071746A* allele in the female gender (Table 3). These differences did not remain significant after multiple comparison analysis applying the false discovery rate correction, with the exception of the SNP *HMOX2* rs1051308 in men (Table 2). The frequencies of *rs2071747* and *rs2270363* did not differ significantly between MS patient and control groups.

Mean ± SD age at onset of MS was similar between MS patients carrying the genotypes *rs2071746AA, rs2071746AT,* and *rs2071746TT* (33.0 ± 12.5, 32.4 ± 10.2, and 32.4 ± 8.9 years, respectively); genotypes *rs2071747GG* and *rs2071747GC* (32.8 ± 11.0 and 30.3 ± 6.2 years, respectively); genotypes *rs2270363GG, rs2270363GA,* and *rs2270363AA* (32.7 ± 11.9, 33.1 ± 9.8, and 29.8 ± 9.1 years, respectively); and genotypes *rs1051308AA, rs1051308AG,* and *rs1051308AA* (32.5 ± 11.6, 33.4 ± 9.9, and 28.6 ± 8.2 years, respectively).

The distribution of the *HMOX1* and *HMOX2* SNPs genotypes and allelic frequencies was similar for those MS patients with “relapsing-remitting”, “primary progressive”, and “secondary progressive” phenotypes of MS, and when compared each type with controls (Table 3).

## Discussion

Data from the present case-control association study suggest a weak association between the allelic variants *HMOX2 rs1051308A* and *HMOX1 rs2071746A* with the risk for MS (although only *HMOX2 rs1051308A* was finally associated after multiple comparison analysis), while the other 2 studied SNPs in the *HMOX* gene were not associated with a modification of MS risk. However, none of the studied SNPs were related with the age at onset of MS or with the specific risk for any of the MS phenotypes.

We have previously reported association between *HMOX1 rs2071746* variant and the risk for PD, ET, and restless legs syndrome^4^,^5^,^7^ suggesting a possible link between these diseases. However, there are no clues on putative biological mechanisms underlying the association found. The rs2071746 SNP is located in the 5′ area, about 500 bp before the coding area, and therefore the most likely mechanism would be related to gene expression. The area where the SNP is located has several transcription factor binding sites. One of these, designated as CUTL1 [T00100] is present in the wild-type sequence, but is disappears in the mutated sequence. The disruption of this transcription factor binding site may underlie differences in terms of gene expression. It has been suggested that both rs2071746 SNP and the *HMOX1* gene promoter (GT)n polymorphism could be related with HMOX1 enzyme activity^20^.

In the brain, the HMOX pathway (in particular HMOX1) is a very important defensive mechanism against oxidative stress, mainly through the degradation of heme to biliverdin, free iron, and CO. Moreover, an up-regulation of HMOX1 expression has been found in the brains of patients with PD, Alzheimer’s disease, and multiple sclerosis.^21^,^22^

A limitation of the current study is that, while 3 of the SNPs studies had a high statistical power, the other one (rs2071747), had not. However, it must be taken in consideration that rs2071747 is an allele with a very low minor frequency (MAF) in healthy Europeans (0.045 in the current study and 0.060 in the 1000 genomes database), and it is very rare in other human populations, with MAF ranging from 0.020 to 0.050 (http://browser.1000genomes.org/Homo_sapiens/Variation/Population? db=core;r=22:35776685-3577785;v=rs2071747;vdb=variation;vf=1641286). Therefore, the minimum sample size to obtain a reliable statistical power for such SNP with a RR value = 1.5 (p = 0.05), is estimated to be 1900 case-control pairs. Moreover, it the significant findings in this study are related to another SNPs that have a relatively high statistical power.

Other potential limitation is the fact that the cohort study included MS patients with different degrees of severity. This does not allow to investigate the influence of *HMOX* genotypes on disability or severity of the disease. The optimum design for this aim should be a prospective one, including the genotyping of patients with a recent diagnosis of MS and the re-examination of the same cohort of MS patients after a similar long-term follow-up period in order to establish the final evolutive type.

Although the results of the present study should be taken with caution because the previously discussed limitations, and deserve a confirmation with further replication studies in other populations, they suggest a slightly increased risk for MS in Spanish Caucasian men carrying the *HMOX2* rs1051308A allele variant, and a trend towards association between the *HMOX1 rs2071746A* and MS risk.

## Methods

### Patients and controls

The study included a total number of 292 MS individuals fulfilling the McDonald’s criteria for definite MS^23^ (91 men, 201 women, mean age 44.1 ± 11.4 years, mean age at onset 32.8 ± 10.9 years; mean expanded disability score scale-EDSS = 3.26 ± 2.39; 157 relapsing-remitting, 93 secondary progressive, and 42 primary progressive MS), who had no other previous neurological diseases, and 533 gender-matched control subjects. Two-hundred and seventy five controls were recruited from the Clínica Universidad de Navarra (Pamplona, Spain) and 258 controls were recruited from the Infanta Cristina University Hospital, (Badajoz, Spain). All consecutive patients diagnosed by consultant neurologists were requested to participate and all of them agreed to do so.

### Ethical aspects

All the participants included in the study gave their written informed consent after full explanation of the procedure. The study, which was conducted in accordance with the principles of the Helsinki declaration of 1975, was approved by the Ethics Committees of Clinical Investigation of the University Hospital “Príncipe de Asturias” (University of Alcalá, Alcalá de Henares, Madrid, Spain), the Infanta Cristina University Hospital (Badajoz, Spain), and Clínica Universitaria de Navarra (Pamplona, Spain). Most of the patients recruited had participated in other previous studies of genetic association with MS risk^1^,^24^,^25^,^26^,^27^,^28^,^29^,^30^.

### Genotyping

Two single nucleotide polymorphisms in the *HMOX1* gene and another two polymorphisms in the *HMOX2* gene were studied using TaqMan probes. These 4 SNPs that were selected because of their putative functional effects and their expected allele frequency in Caucasian individuals^4^,^6^. *HMOX1* SNP rs2071746 (an upstream variant), *HMOX1* rs2071747 (a missense mutation within the exon 1 of the *HMOX1* gene), the SNP rs2270363 (a polymorphism in the regulatory region of the human *HMOX2* gene), and rs1051308 (a polymorphism in the 3′ untranslated region).

Genotyping, which was performed in genomic DNA from venous blood samples of participants, was carried out using TaqMan assays (Life Technologies, Alcobendas, Madrid, Spain) designed to detect the four previously mentioned SNPs designated respectively by the supplier with the following part numbers: C__15869717_10, C__22272778_10, C__15957370_10 and C___9695078_1_. An Eppendorf realplex thermocycler, using fluorescent probes, was used for the detection by qPCR. The amplification conditions were the following: after a denaturation time of 10 min at 96 °C, 45 cycles of 92 °C 15 sec and 60 °C 90 sec were carried out and fluorescence was measured at the end of each cycle and at endpoint. Determinations were done by triplicate in all samples, and then genotypes were assigned both using a gene identification software (RealPlex 2.0, Eppendorf) and analysing the reference cycle number for each fluorescence curve, calculated by means of the CalQPlex algorithm (Eppendorf).

Copy number variations (CNVs) of the *HMOX1* and *HMOX2* genes were analyzed using the TaqMan copy number assays Hs00774483_cn and Hs01223070_cn, respectively. Both assays were designed to hybridize within the open reading frame in the target genes (Life Technologies, Alcobendas, Madrid, Spain). Amplification was carried out in an Applied Biosystems 7500 real-time thermocycler as described by the manufacturer, using as a copy number reference assay RNAse P. All reactions were carried out in quadruplicate. Results were analyzed by means of the CopyCaller Software (Life Technologies, Alcobendas, Madrid, Spain). According to standard procedures in CNV analyses, we designed as heterozygous (null/present) those samples with a single copy of the corresponding gene. Since the probes were designed to detect exonic sequences, even if the rest of the gene would remain in these so called null alleles, the translated protein would not be functional.

### Statistical analysis

The DeFinetti program (http://ihg.gsf.de/cgi-bin/hw/hwa1.pl), the PLINK software^31^, and the program PHASE v2.1.1^32^ were used, respectively, to analyze the Hardy-Weinberg equilibrium, to perform the allelic and genotype analyses, and to perform haplotype reconstruction using the default model for recombination rate variation with 1000 iterations, 500 burn-in iterations, and a thinning interval of 1. Diplotypes were obtained from the combination of haplotypes in the best run (the one that showed the maximum consistency of results across all runs^33^ Statistical analyses were performed using the SPSS 19.0 for Windows (SPSS Inc., Chicago, Illinois, USA). We calculated intergroup comparison values by means of the χ2 or Fisher tests when appropriate, and calculated the 95% confidence intervals as well. We used the False discovery rate procedure^34^ to calculate correction for multiple testing (Pc values).

The determination of the sample size was done from variant allele frequencies observed in control individuals with a genetic model analyzing the frequency for carriers of the disease gene with a RR value = 1.5 (p = 0.05). The statistical power for two-tailed associations for the presence of the SNPs identified in this study (rs2071746, rs2071747, rs2270363 and rs1051308) was 97.6%, 43.1%, 96.3% and 97.0%. Testing for heterogeneous association (homogeneity test) was analyzed by using the Breslow–Day test.

The negative predictive value (NPV) was calculated as d/r2 (d = number of control individuals with the risk factor absent; r2 = sum of patients and controls with the risk factor absent)^35^.

## Additional Information

**How to cite this article**: Agúndez, J. A. G. *et al. Heme Oxygenase-1 And 2 Common* Genetic Variants And Risk For Multiple Sclerosis. *Sci. Rep.* **6**, 20830; doi: 10.1038/srep20830 (2016).

## Figures and Tables

**Table 1:** *HMOX* genotypes and allelic variants of patients with MS and healthy volunteers.

GENOTYPES	MS PATIENTS (N = 292, 584 alleles)	CONTROLS (N = 533, 1066 alleles)	OR (95% CI), P; Pc; NPV (95% CI)
HMOX1 rs2071746 A/A	102 (34.9; 29.5–40.4)	154 (28.9; 25.0–32.7)	1.32 (0.96–1.81); 0.073; 0.292; 0.67 (0.64–0.69)
A/T	139 (47.6; 41.9–53.3)	256 (48.0; 43.8–52.3)	0.98 (0.73–1.32); 0.907; 0.973; 0.64 (0.61–0.68)
T/T	51 (17.5; 13.1–21.8)	122 (22.9; 19.3–26.5)	0.71 (0.49–1.04); 0.067; 0.292; 0.63 (0.61–0.65)
Null/A	0 (—)	1 (0.2; 0.-2-0.6)	0.00 (0.00–31.69); 0.459; 0.667; 0.65 (0.65-0.65)
Null/T	0 (—)	0 (—)	—
HMOX1 rs2071747 G/G	266 (91.1; 87.8–94.4)	485 (91.0; 88.6–93.4)	1.01 (0.60–1.72); 0.961; 0.973; 0.65 (0.53–0.75)
G/C	25 (8.6; 5.4–11.8)	46 (8.6; 6.2–11.0)	0.99 (0.58–1.70); 0.973; 0.973; 0.65 (0.64–0.66)
C/C	1 (0.3; 0.-3-1.0)	1 (0.2; 0.-2-0.6)	1.82 (0.05–67.02); 0.666; 0.819; 0.65 (0.65–0.65)
Null/G	0 (—)	1 (0.2; 0.-2-0.6)	0.00 (0.00–31.69); 0.459; 0.667; 0.65 (0.65–0.65)
Null/C	0 (—)	0 (—)	—
HMOX2 rs2270363 G/G	127 (43.5; 37.8–49.2)	253 (47.5; 43.2–51.7)	0.85 (0.63–1.15); 0.274; 0.667; 0.63 (0.60–0.66)
G/A	130 (44.5; 38.8–50.2)	227 (42.6; 38.4–46.8)	1.08 (0.80–1.46); 0.593; 0.790; 0.65 (0.62–0.68)
A/A	35 (12.0; 8.3–15.7)	52 (9.8; 7.2–12.3)	1.26 (0.78–2.03); 0.319; 0.667; 0.65 (0.64–0.66)
Null/G	0 (—)	1 (0.2; 0.-2-0.6)	0.00 (0.00–31.69); 0.459; 0.667; 0.65 (0.65–0.65)
Null/A	0 (—)	0 (—)	—
HMOX 2 rs1051308 A/A	147 (50.3; 44.6–56.1)	227 (42.6; 38.4–46.8)	1.37 (01.02–0.84); 0.033; 0.292; 0.68 (0.65–0.71)
A/G	120 (41.1; 35.5–46.7)	236 (44.3; 40.1–48.5)	0.88 (0.65–1.19); 0.378; 0.667; 0.63 (0.60–0.66)
G/G	25 (8.6; 5.4–11.8)	69 (12.9; 10.1–15.8)	0.63 (0.68–1.04); 0.058; 0.292; 0.64 (0.62–0.65)
Null/A	0 (—)	0 (—)	—
Null/G	0 (—)	1 (0.2; 0.-2-0.6)	0.00 (0.00–31.69); 0.459; 0.667; 0.65 (0.65–0.65)
ALLELES			
HMOX1 rs2071746 A	343 (58.7; 54.7–62.7)	565 (53.0; 50.0–56.0)	1.26 (1.02–1.56); 0.025; 0.063; 0.68 (0.65–0.70)
T	241 (41.3; 37.3–45.3)	501 (47.0; 44.0–50.0)	0.79 (0.64–0.98); 0.025; 0.063; 0.62 (0.60–0.64)
HMOX1 rs2071747 G	557 (95.4; 93.7–97.1)	1017 (95.5; 94.2–96.7)	0.97 (0.59–1.62); 0.914; 0.914; 0.64 (0.52–0.74)
C	27 (4.6; 2.9–6.3)	48 (4.5; 3.3–5.8)	1.03 (0.62–1.71); 0.914; 0.914; 0.65 (0.64–0.65)
HMOX2 rs2270363 G	384 (65.8; 61.9–69.6)	734 (68.9; 66.1–71.7)	0.87 (0.70–1.08); 0.188; 0.313; 0.62 (0.59–0.66)
A	200 (34.2; 30.4–38.1)	331 (31.1; 28.3–33.9)	1.16 (0.93–1.44); 0.188; 0.313; 0.66 (0.64–0.67)
HMOX 2 rs1051308 A			
	414 (70.9; 67.2–74.6)	690 (64.8; 61.9–67.7)	1.32 (1.06–0.66); 0.012; 0.060; 0.69 (0.65–0.72)
G	170 (29.1; 25.4–32.8)	375 (35.2; 32.3–38.1)	0.76 (0.60–0.95); 0.012; 0.060; 0.63 (0.61–0.64)
Null HMOX1	0 (—)	1 (0.1; 0.-1-0.3)	0.00 (0.00–31.61); 0.459; 0.573; 0.65 (0.65–0.65)
Null HMOX 2	0 (—)	1 (0.1; 0.-1-0.3)	0.00 (0.00–31.61); 0.459; 0.573; 0.65 (0.65–0.65)

The values in each cell represent: number (percentage; 95% confidence intervals). Pc probability after correction for multiple comparisons. NPV: negative predictive value.

**Table 2:** *HMOX* genotypes and allelic variants of patients with MS and healthy volunteers distributed by gender.

GENOTYPES	MS WOMEN (N = 201, 402 ALLELES)	CONTROL WOMEN (N = 367, 734 ALLELES)	INTERGROUP COMPARISON OR (95% CI), P; PC ; NPV (95% CI)	MS MEN (N = 91, 182 ALLELES)	CONTROL MEN (N = 165, 332 ALLELES)	INTERGROUP COMPARISON OR (95% CI) P; PC; NPV (95% CI)
HMOX1 rs2071746 A/A	69 (34.3; 27.8–40.9)	101 (27.5; 23.0–32.1)	1.38 (0.93–2.03); 0.091; 0.396; 0.67 (0.640.70)	33 (36.3; 26.4–46.1)	53 (32.1; 25.0–39.2)	1.20 (0.68–2.13); 0.503; 0.721; 0.66 (0.62–0.70)
A/T	98 (48.8; 41.8–55.7)	182 (49.6; 44.5–54.7)	0.97 (0.68–1.39); 0.849; 0.858; 0.64 (0.60–0.68)	41 (45.1; 34.8–55.3)	74 (44.8; 37.3–52.4)	1.01 (0.58–1.74); 0.975; 0.975; 0.65 (0.59–0.70)
T/T	34 (16.9; 11.7–22.1)	84 (22.9; 18.6–27.2)	0.69 (0.43–1.09); 0.094; 0.396; (0.63 (0.61–0.65)	17 (18.7; 10.7–26.7)	38 (23.0; 16.6–29.5)	0.77 (0.39–1.52); 0.418; 0.721; 0.63 (0.60–0.67)
HMOX1 rs2071747 G/G	181 (90.0; 85.9–94.2)	334 (91.0; 88.1–93.9)	0.89 (0.48–1.67); 0.708; 0.858; 0.62 (0.49–0.75)	85 (93.4; 88.3–98.5)	151 (91.5; 87.3–95.8)	1.31 (0.45–4.00); 0.590; 0.721; 0.70 (0.47–0.87)
G/C	19 (9.5; 5.4–13.5)	32 (8.7; 5.8–11.6)	1.09 (0.58–2.06); 0.770; 0.858; 0.65 (0.64–0.66)	6 (6.6; 1.5–11.7)	14 (8.5; 4.2–12.7)	0.76 (0.25–2.22); 0.590; 0.721; 0.64 (0.63–0.66)
C/C	1 (0.5; 0.-5-1.5)	1 (0.3; 0.3–0.8)	1.83 (0.05–67.24); 0.665; 0.858; 0.65 (0.65–0.65)	0 (0.0; 0.0-0.0)	0 (0.0; 0.0-0.0)	—
HMOX2 rs2270363 G/G	85 (42.3; 35.5–49.1)	175 (47.7; 42.6–52.8)	0.80 (0.56–1.15); 0.218; 0.654; 0.62 (0.59–0.66)	42 (46.2; 35.9–56.4)	78 (47.3; 39.7–54.9)	0.96 (0.55–1.65); 0.864; 0.950; 0.64 (0.58–0.70)
G/A	87 (43.3; 36.4–50.1)	156 (42.5; 37.4–47.6)	1.03 (0.72–1.48); 0.858; 0.858; 0.65 (0.61–0.69)	43 (47.3; 37.0–57.5)	71 (43.0; 35.5–50.6)	1.19 (0.69–2.05); 0.516; 0.721; 0.66 (0.61–0.72)
A/A	29 (14.4; 9.6–19.3)	36 (9.8; 6.8–12.9)	1.55 (0.89–2.70); 0.099; 0.396; 0.66 (0.64–0.67)	6 (6.6; 1.5–11.7)	16 (9.7; 5.2–14.2)	0.66 (0.22–1.88); 0.397; 0.721; 0.64 (0.62–0.66)
HMOX 2 rs1051308 A/A	94 (46.8; 39.9–53.7)	158 (43.1; 38.0–48.1)	1.16 (0.81–1.67); 0.395; 0.858; 0.66 (0.63–0.70)	53 (58.2; 48.1–68.4)	69 (41.8; 34.3–49.3)	1.94 (0.12–3.37); 0.012; 0.072; 0.72 (0.66–0.77)
A/G	85 (42.3; 35.5–49.1)	161 (43.9; 38.8–48.9)	0.94 (0.65–1.35); 0.716; 0.858; 0.64 (0.60–0.68)	35 (38.5; 28.5–48.5)	75 (45.5; 37.9–53.1)	0.75 (0.43–1.31); 0.280; 0.721; 0.62 (0.56–0.67)
G/G	22 (10.9; 6.6–15.3)	48 (13.1; 9.6–16.5)	0.82 (0.46–1.44); 0.460; 0.858; 0.64 (0.63–0.66)	3 (3.3; 0.–4–7.0)	21 (12.7; 7.6–17.8)	0.23 (0.06–0.86); 0.013; 0.072; 0.62 (0.61–0.64)
ALLELES						
HMOX1 rs2071746 A	236 (58.7; 53.9–63.5)	384 (52.3; 48.7–55.9)	1.30 (1.01–1.67); 0.039; 0.156; 0.68 (0.65–0.71)	107 (58.8; 51.6–65.9)	180 (54.5; 49.2–59.9)	1.19 (0.81–1.74); 0.355; 0.710; 0.67 (0.62–0.72)
T	166 (41.3; 36.5–46.1)	350 (47.7; 44.1–51.3)	0.77 (0.60–0.99); 0.039; 0.156; 0.62 (0.59–0.65)	75 (41.2; 34.1–48.4)	150 (45.5; 40.1–50.8)	0.84 (0.57–1.23); 0.355; 0.710; 0.63 (0.59–0.67)
HMOX1 rs2071747 G	381 (94.8; 92.6–97.0)	700 (95.4; 93.8–96.9)	0.88 (0.49–1.60); 0.657; 0.657; 0.62 (0.48–0.74)	176 (96.7; 94.1–99.3)	316 (95.8; 93.6–97.9)	1.30 (0.46–3.86); 0.597; 0.796; 0.70 (0.46–0.87)
C	21 (5.2; 3.0–7.4)	34 (4.6; 3.1–6.2)	1.14 (0.63–2.05); 0.657; 0.657; 0.65 (0.64–0.66)	6 (3.3; 0.7–5.9)	14 (4.2; 2.1–6.4)	0.77 (0.26–2.19); 0.597; 0.796; 0.64 (0.64–0.65)
HMOX2 rs2270363 G	257 (63.9; 59.2–68.6)	506 (68.9; 65.6–72.3)	0.80 (0.61–1.04); 0.086; 0.172; 0.61 (0.57–0.65)	127 (69.8; 63.1–76.5)	227 (68.8; 63.8–73.8)	1.05 (0.69–1.58); 0.816; 0.816; 0.65 (0.59–0.72)
A	145 (36.1; 31.4–40.8)	228 (31.1; 27.7–34.4)	1.25 (0.96–1.63); 0.086; 0.172; 0.66 (0.64–0.68)	55 (30.2; 23.5–36.9)	103 (31.2; 26.2–36.2)	0.95 (0.63–1.44); 0.816; 0.816; 0.64 (0.61–0.67)
HMOX 2 rs1051308 A	273 (67.9; 63.3–72.5)	477 (65.0; 61.5–68.4)	1.14 (0.87–1.49); 0.320; 0.427; 0.67 (0.63–0.71)	141 (77.5; 71.4–83.5)	213 (64.5; 59.4–69.7)	1.89 (1.22–2.92); 0.002; 0.008; 0.74 (0.68–0.80)
G	129 (32.1; 27.5–36.7)	257 (35.0; 31.6–38.5)	0.88 (0.67–1.15); 0.320; 0.427; 0.64 (0.62–0.66)	41 (22.5; 16.5–28.6)	117 (35.5; 30.3–40.6)	0.53 (0.34–0.82); 0.002; 0.008; 0.60 (0.58–0.63)

The values in each cell represent: number (percentage; 95% confidence intervals). Pc probability after correction for multiple comparisons. NPV: negative predictive value.

Control individuals with null genotypes were excluded because copy number variations were not relevant as putative risk factors (see above).

**Table 3:** *HMOX* genotypes and allelic variants of patients with MS according with the evolutive type of MS.

	Relapsing-remitting MS No (%; 95% CI)	Secondary progressive No (%; 95% CI)	Primary progressive No (%; 95% CI)
GENOTYPES			
HMOX1 rs2071746 A/A	55 (35.0; 27.6–42.5)	29 (31.2; 21.8–40.6)	18 (42.9; 27.9–57.8)
A/T	74 (47.1; 39.3–54.9)	47 (50.5; 40.4–60.7)	18 (42.9; 27.9–57.8)
T/T	28 (17.8; 11.8–23.8)	17 (18.3; 10.4–26.1)	6 (14.3; 3.7–24.9)
HMOX1 rs2071747 G/G	145 (92.4; 88.2–96.5)	85 (91.4; 85.7–97.1)	36 (85.7; 75.1–96.3)
G/C	11 (7.0; 3.0–11.0)	8 (8.6; 2.9–14.3)	6 (14.3; 3.7–24.9)
C/C	1 (0.6; 0.-6-1.9)	0 (0.0; 0.0-0.0)	0 (0.0; 0.0-0.0)
HMOX2 rs2270363 G/G	70 (44.6; 36.8–52.4)	43 (46.2; 36.1–56.4)	14 (33.3; 19.1–47.6)
G/A	65 (41.4; 33.7–49.1)	39 (41.9; 31.9–52.0)	26 (61.9; 47.2–76.6)
A/A	22 (14.0; 8.6–19.4)	11 (11.8; 5.3–18.4)	2 (4.8; -1.-7-11.2)
HMOX 2 rs1051308 A/A	78 (49.7; 41.9–57.5)	48 (51.6; 41.5–61.8)	21 (50.0; 34.9–65.1)
A/G	64 (40.8; 33.1–48.5)	36 (38.7; 28.8–48.6)	20 (47.6; 32.5–62.7)
G/G	15 (9.6; 5.0–14.2)	9 (9.7; 3.7–15.7)	1 (2.4; -2.-2-7.0)
ALLELES			
HMOX1 rs2071746 A	184 (58.6; 53.2–64.0)	105 (56.5; 49.3–63.6)	54 (64.3; 54.0–74.5)
T	130 (41.4; 36.0–46.8)	81 (43.5; 36.4–50.7)	30 (35.7; 25.5–46.0)
HMOX1 rs2071747 G	301 (95.9; 93.7–98.1)	178 (95.7; 92.8–98.6)	78 (92.9; 87.3–98.4)
C	13 (4.1; 1.9–6.3)	8 (4.3; 1.4–7.2)	6 (7.1; 1.6–12.7)
HMOX2 rs2270363 G	205 (65.3; 60.0–70.6)	125 (67.2; 60.5–74.0)	54 (64.3; 54.0–74.5)
A	109 (34.7; 29.4–40.0)	61 (32.8; 26.0–39.5)	30 (35.7; 25.5–46.0)
HMOX 2 rs1051308 A	220 (70.1; 65.0–75.1)	132 (71.0; 64.4–77.5)	62 (73.8; 64.4–83.2)
G	94 (29.9; 24.9–35.0)	54 (29.0; 22.5–35.6)	22 (26.2; 16.8–35.6)

The values in each cell represent: number (percentage; 95% confidence intervals).

None of the subgroups of MS patients displayed statistically significant differences as compared to control individuals.
